# On the maximal cliques in *c*-max-tolerance graphs and their application in clustering molecular sequences

**DOI:** 10.1186/1748-7188-1-9

**Published:** 2006-05-31

**Authors:** Katharina A Lehmann, Michael Kaufmann, Stephan Steigele, Kay Nieselt

**Affiliations:** 1 Parallel Computing, Wilhelm-Schickard-lnstitut, University of Tuebingen, Sand 14, D-72076 Tuebingen, Germany; 2 Center for Bioinformatics Tuebingen, Wilhelm-Schickard-lnstitut, University of Tuebingen, Sand 14, D-72076 Tuebingen, Germany

## Abstract

Given a set *S *of *n *locally aligned sequences, it is a needed prerequisite to partition it into groups of very similar sequences to facilitate subsequent computations, such as the generation of a phylogenetic tree. This article introduces a new method of clustering which partitions *S *into subsets such that the overlap of each pair of sequences within a subset is at least a given percentage *c *of the lengths of the two sequences. We show that this problem can be reduced to finding all maximal cliques in a special kind of max-tolerance graph which we call a *c-max-tolerance *graph. Previously we have shown that finding all maximal cliques in general max-tolerance graphs can be done efficiently in *O*(*n*^3 ^+ *out*). Here, using a new kind of sweep-line algorithm, we show that the restriction to *c*-max-tolerance graphs yields a better runtime of *O*(*n*^2 ^log *n *+ *out*). Furthermore, we present another algorithm which is much easier to implement, and though theoretically slower than the first one, is still running in polynomial time. We then experimentally analyze the number and structure of all maximal cliques in a *c*-max-tolerance graph, depending on the chosen *c*-value. We apply our simple algorithm to artificial and biological data and we show that this implementation is much faster than the well-known application Cliquer. By introducing a new heuristic that uses the set of all maximal cliques to partition *S*, we finally show that the computed partition gives a reasonable clustering for biological data sets.

## 1 Introduction

**Figure 1:**
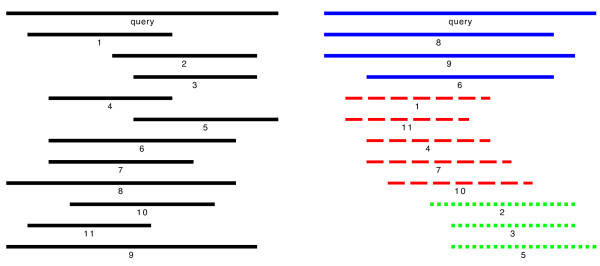
The left side shows a set of 11 sequences that are aligned to a common reference sequence, but do not have any special ordering that makes it easy to distinguish clusters of similar sequences. On the right a reasonable ordering is given where colors indicate groups of sequences that have a 60% overlap for each pair of sequences. Note that the given coloring might not be the only reasonable.

Viewing the subject sequences aligned to a query sequence that result from a BLAST-based [1] comparison, in many cases one can identify groups of sequences clustering around different subintervals of the query sequence. Often, the decision by eye to which cluster a certain sequence belongs, is strongly depending on the order in which the sequences are presented. Fig. 1a) shows a schematic sketch of aligned sequences in random order. The sequences seem to form two, or maybe three groups. The same sequences in Fig. 1b) are ordered according to how many positions they have in common and colors indicate those sequences that share a large part of their sequence. The algorithm finds three different clusters of sequences. A cluster in this sense can be defined as a subset of aligned sequences that have approximately the same length and that are aligned to approximately the same subinterval of the query sequence. As we have argued above, the human eye may be fooled by the ordering of the presented sequences and humans are limited in the number of sequences they can group into clusters, and thus the automatic and objective computation of a clustering is an important task.

**Figure 2:**
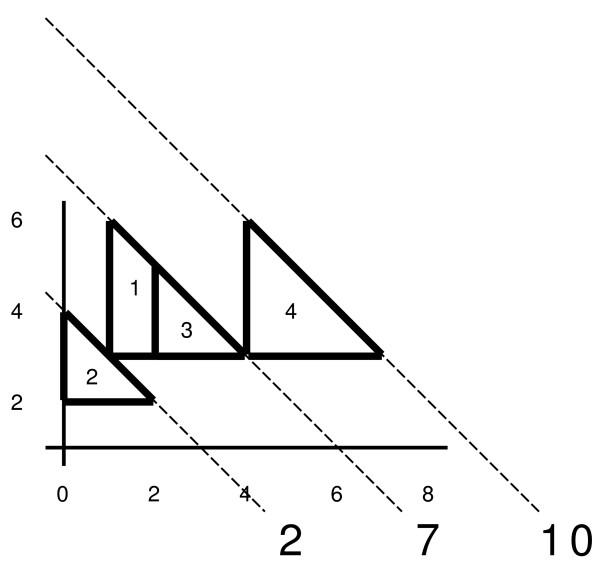
The semi-squares can be ordered according to the height of their hypotenuse at a given *x*-position.

One kind of clustering can be obtained by using BlastClust from the NCBI-BLAST package [2]. It is a clustering tool designed especially to cluster protein or DNA sequences based on pairwise matches returned by the BLAST algorithm. It uses BLAST scores to assign statistical significance, matches pairs that reach that level of significance, then constructs clusters using a simple, greedy, single-linkage clustering method. However, empirically it is known that BlastClust is very limited with respect to the size of the input set. Furthermore, in many instances, the application of this heuristic is also limited by the fact that one unique clustering does not exist and that in fact many clusterings are possible and reasonable for different applications: for example, when annotating putative domains of a protein sequence, it is important to find common regions that are shared by large groups of sequences, while for the computation of phylogenetic trees it is important to find groups that share the largest possible part of their sequences.

In any case, under a given constraint, the groups should be as large as possible. Both constraints can be formulated as follows: Given two sequences, they are said to *tolerate *each other if both overlap at least an absolute amount *t*, the *tolerance*, or a *relative tolerance c*% of the length of the other sequence. This leads naturally to the family of *max-tolerance graphs *[3], where vertices represent intervals, and two vertices are connected by an edge if the corresponding sequences tolerate each other. The question for maximal clusters now reduces to finding maximal cliques in the *max-tolerance graph*.

Interestingly, the history of a special max-tolerance graph with *t *= 1 is deeply intertwined with the study of the DNA: Benzer was the first to raise the question, whether the sub-elements of the DNA are arranged linearly, or not [4]. To answer this question, sequences of DNA are represented as vertices, where two vertices are connected by an edge if the corresponding sequences are overlapping on at least one position. The resulting graph is an *interval graph *[5,6]. It could be shown that a graph generated in this way can only be derived from fragments of a *linear *sequence if it has a certain property. Since the overlapping graph derived from DNA fragments showed this linearity property, this was yet another confirmation that the genome of organisms consists of long, linear DNA molecules. Searching for maximal cliques in interval graphs can be done efficiently in time linear to the size of the graph. However, for computing meaningful biological clusters, an absolute tolerance of *t *= 1 is not appropriate. For this purpose, it is necessary to increase the *tolerance *to a reasonable value and in addition to use the relative tolerance *c *of the length of the sequences. Thus, for *c *= 0.5 two sequences will tolerate each other if both have approximately the same length and share half of their sequence, or if one is not longer than twice the length of the other and overlaps the second completely, and anything in between. We call this kind of *max-tolerance graph *with a fixed relative tolerance *c *for all sequences a *c-max-tolerance graph*. The question of finding maximal groups of sequences that pairwise tolerate each other now reduces to the question of finding all maximal cliques, in the respective *c*-max-tolerance graph.

For general graphs, the computation of all maximal cliques is an NP-hard problem, since it can be reduced to the maximum clique problem which is again a classical NP-complete graph problem [7]. A well-known and popular branch-and-bound based software to compute maximum and maximal cliques in general graphs is Cliquer [8]. Cliquer's runtime is exponential. It will turn out that the maximal clique problem for the graphs considered here is not NP-hard.

Our aim here is now to show that, based on results of general max-tolerance graphs, an efficient algorithm can be applied to find all maximal cliques in a *c*-max-tolerance graph. In addition, we show how this set of all maximally cliques can then be used to find reasonable clusterings for biological sequences aligned to each other.

Focusing on the biological application of the idea of *c*-max-tolerance graphs, our approach is two-fold: First, we will give a practical algorithm to report all maximal cliques in polynomial time. This algorithm is conceptually simple, thus ensuring that it is easy to implement it correctly and numerically stable. We will also show in the experimental section that its implementation is very fast for typical data sizes in biological applications. Second, based on the general principle of the simple algorithm, we will present a more involved, output-sensitive algorithm in *O*(*n*^2 ^log *n *+ *out*), where *out *denotes the size of the output. This is a considerable improvement compared with the corresponding bound for general max-tolerance graphs obtained in [9].

With this two-fold approach we follow the ideas of the field of algorithm design [10], that stresses the point that an easy algorithm should always be preferred for an implementation unless the data is so big that the more efficient algorithm has to be used.

The result of the algorithm can then be used to cluster the data in an automated and objective fashion. We are facing two problems here: First, the number of maximal cliques is strongly depending on the chosen *c*-value. Second, for many instances most of the sequences are elements of more than one maximal clique. Thus, we will give a heuristic based on the set of all maximal cliques that is able to find a reasonable clustering. We illustrate the algorithm using artificial as well as three different biological examples. The first of the biological data sets is an example of a repeat analysis, where a transposon from the nematode *Pristionchus pacificus *is compared with reads of BAC clone data covering about half of the genome of that organism. The second and third example are proteins that are compared with the UniProt database [11]. The article is structured as follows: in Section 2 we give a short review on results that are essential for the understanding of the two algorithms together with required definitions. Section 3 presents the first, simpler algorithm, followed by the description of the algorithm with a new, lower runtime complexity for *c*-max-tolerance graphs in Section 4. Section 5 gives some results on experiments and Section 6 summarizes and discusses our results.

## 2 Mathematical background

In this section we will define the formal basis and repeat some earlier derived results that are essential for the understanding of the rest of the article.

Let *S *denote a set of *n *intervals *I*_*i *_= [*x*_*i*_, *y*_*i*_], 1 ≤ *i *≤ *n*, *x*_*i*_, *y*_*i *_∈ ℕ, *x*_*i *_<*y*_*i*_, where *x*_*i *_denotes the coordinate of the left endpoint and *y*_*i *_denotes the coordinate of the right endpoint. The length |*I*_*i*_| of an interval is defined as *y*_*i *_- *x*_*i*_. Let *I*_*i *_= [*x*_*i*_, *y*_*i*_] and *I*_*j *_= [*x*_*j*_, *y*_*j*_] be two intervals. Their *overlap length *|*I*_*i *_⋂ *I*_*j*_| is given by:

|*I*_*i *_⋂ *I*_*j*_| = min{*y*_*i*_, *y*_*j*_} - max{*x*_*i*_, *x*_*j*_}.

A *c*-max-tolerance graph *G *= (*V, E*) is associated with a tolerance parameter 0 ≤ c ≤ 1 and it is then given by *V *= {*v*_1_, ...,*v*_*n*_} representing the set of intervals and

*E *= {{*v*_*i*_, *v*_*j*_}||*I*_*i *_⋂ *I*_*j*_| ≥ *c *· max{|*I*_*i*_|, |*I*_*j*_|}}       (1)

for 1 ≤ *i,j *≤ *n*. Pairs of intervals satisfying this condition are said to *tolerate each other*. More generally, interval *I*_*i *_*tolerates *interval *I*_*j *_if |*I*_*i *_⋂ *I*_*j*_| > *c *· |*I*_*i*_|.

In a general *max-tolerance graph *each interval *I*_*i *_is associated with a *tolerance *0 ≤ *t*_*i *_≤ |*I*_*i*_| and here, two intervals are said to *tolerate each other*, if |*I*_*i *_⋂ *I*_*j*_*| *≥ max{*t*_*i*_, *t*_*j*_}.

Given a graph *G *= (*V, E*), a *clique *is a subset *C *⊆ *V *of pairwise-connected vertices. A clique *C *is called a *maximal clique *if there is no other clique *C' *⊃ *C*. A clique is a *maximum clique *if its cardinality is of largest possible size.

The following lemma has been derived for general max-tolerance graphs and applies also to *c*-max-tolerance graphs [9]:

**Lemma 1 ***The number of maximal cliques in (c-)max-tolerance graphs is O*(*n*^3^).

This result has been derived by a fundamental equivalence, for which we will need the following definitions: Let *S *= {[*x*_1_, *y*_1_], [*x*_2_, *y*_2_],..., [*x*_*n*_, *y*_*n*_]} be a set of intervals and let 0 <*c *≤ 1.0 denote the same relative tolerance for all intervals. Let *T*(*S*) denote a set of triangles Δ_*i *_= {*A*_*i*_, *B*_*i*_, *C*_*i*_}, *A*_*i*_, *B*_*i*_, *C*_*i *_∈ (ℕ × ℕ) with the following coordinates:

*A*_*i *_= (*x*_*i*_, |*I*_*i*_| - ⌊|*I*_*i*_| * (1 - *c*)⌋)       (2)

*B*_*i *_= (*x*_*i *_+ ⌊|*I*_*i*_| * (1 - *c*)⌋, |*I*_*i*_| - ⌊|*I*_*i*_| * (1 - *c*)⌋)       (3)

*C*_*i *_= (*x*_*i*_, |*I*_*i*_|)       (4)

Thus, these triangles look like squares of length ⌊|*I*_*i*_| * (1 - *c*)⌋, located at (*x*_*i*_, ⌊|*I*_*i*_| * (1 - *c*)⌋), cut in half by the diagonal from their upper left to their lower right corner. Therefore, we will call these triangles *semi-squares*. The next lemma states the following equivalence:

**Lemma 2 ***The c-max-tolerance graph of *(*S,c*) *is equivalent to the intersection graph of T*(*S*).

**Figure 3:**
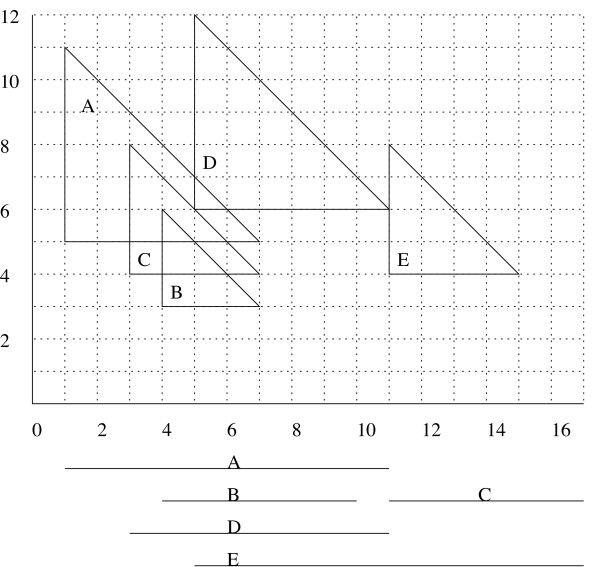
A set of intervals **A-E **together with its corresponding semi-square representation. This example for *c *= 0.5 also shows that not all candidate cliques are maximal with respect to the set of active semi-squares: When *D *is born, there are two maximal cliques in the *y*-structure: {*A, B*} and {*B, C*}. *D *is intersecting with *A *and *B*. Thus, there will be one candidate clique {*A, B, D*}, built from clique {*A, B*}, and {*A, D*}, built from {*B, C*}. The latter candidate clique is thus not maximal with respect to all active cliques and should not be inserted into *Q*.

The lemma states that an edge between two intervals is drawn if and only if their corresponding semi-squares intersect. The formal proof is given in [9] but we want to motivate it here: an edge between two intervals *A *and *B *is drawn if they tolerate each other. It follows, that *A *can tolerate *B *if *B *does not start right of ⌊|*A*| (1 - *c*)⌋; otherwise |*A *⋂ *B *<*c *· |*A*|. Note that the *y*-coordinate of a semi-square corresponding to an interval is equal to ⌊|*A*| (1 - *c*)⌋ and that the semi-square ends at *x*_*A *_+ ⌊|*A*| (1 - *c*)⌋. On the other hand, any overlap with *A *cannot be longer than |*A*|, and this only in the case where *B *fully overlaps *A*. If *B *starts at *x*_*A *_+ 1, their overlap can be of length at most |*A*| - 1, etc. The semi-square illustrates exactly this: The *y*-coordinate of the hypotenuse of any semi-square at position *x *gives a bound on the length of an overlap the corresponding interval can have with any other interval starting at *x*. Thus, two semi-squares will intersect if and only if the corresponding intervals tolerate each other: Let *A *be the longer interval, i.e., it determines the minimal length of the intersection, denoted by the *y*-coordinate of the basis of its corresponding semi-square. This semi-square can only be intersected by those semi-squares whose basis are below that of *A *and have a hypotenuse that is not lower than the basis of *A *at least at some point within the interval of *A*. Fig. 3 shows a set of intervals and their corresponding set of semi-squares.

The semi-squares can be ordered according to three different criteria: either by their left sides *x*_*i*_, or their *y*-coordinate of their basis at |*I*_*i*_| - ⌊|*I*_*i*_| * (1 - *c*)⌋ or by their hypotenuse. Hypotenuses can be easily ordered according to their height at a given *x*-position by noting that the slope is -1 for all of them. Thus, for every *x*-coordinate with *x *between *x*_*i *_and *x*_*i *_+ ⌊|*I*_*i*_| * (1 - *c*)⌋, the following equation is valid:

*const *= *y*_*i *_- (*x *- *x*_*i*_)       (5)

where *y*_*i *_is the endpoint of the corresponding interval *I*_*i*_. Thus, for a given *x*-position the height of the hypotenuse of each active semi-square is determined by *y*_*i *_+ *x*_*i *_- *x *(s. Fig. 2).

Since *x *is the same for all active semi-squares we can order the semi-squares by *y*_*i *_+ *x*_*i*_. An important observation is that the order by the *y*-coordinate of the basis of the semi-squares or by their hypotenuse is not the same in general.

A very general tool in geometric algorithms is that of a *sweep-line *[12]: Here, so called *events *are ordered and then processed in this order, thus, we process the events in a *sweep*, where the invariant is that all events up until a certain point in the ordered list are already processed and that the events after this point still have to be processed. In geometrical problems such as finding the intersection points of a set of lines, an *event *is typically something as the begin or *birth *of a geometric structure and the end or *death *of it.

## 3 Simple algorithm for computing all maximal cliques in *c*-max-tolerance graphs

The sweep-line algorithm presented in this section is based on the semi-square intersection graph corresponding to a given *c*-max-tolerance graph. We will show that the theoretical runtime is a bit higher than the one given in the following section, but this algorithm is very easy to implement and it will lay the conceptual basis for the more involved algorithm presented in the next section. We will show in Section 5 that the implementation based on this simpler algorithm is nonetheless very fast, it will compute all maximal cliques in real data sets within a few seconds. The virtual sweep is done along the *x*-axis, handling two kinds of events: The *birth *of semi-squares and their *death*, i.e., start and end of a semi-square, respectively. As long as the sweep handles events with an *x*-position within the basis of a semi-square *X, X *is said to be *active*. The so-called *y*-structure *Q *holds all maximal cliques of active members at any time and every maximal clique is contained only once (*y-structure invariant*), where maximality refers only to the set of **active **semi-squares and not necessarily to the set of all semi-squares. In the first step all *x*-events are sorted non-decreasingly according to their *x*-position. If there are death and birth events at the same *x*-position, the birth events are first inserted into the sorted list. Then, the events are extracted from the sorted list which enables the virtual *x*-axis sweep.

For every **birth event**, every maximal clique *C *in the *y*-structure is checked, whether some of the semi-squares are intersecting with the newborn semi-square *X*. We have to differentiate the following cases:

1. The handling is straightforward if *X *intersects with all semi-squares in the clique. Then, *X *can just be added to the clique.

2. For every clique where at least one but not all semi-squares are intersecting *X*, a candidate clique is built with all intersecting semi-squares together with the newborn semi-square. This operation is called the *splitting of a clique*. It is important to note that not all candidate cliques are really maximal, as is illustrated in Fig. 3. To maintain the *y*-structure invariant, it is important to insert only cliques that are maximal with respect to the set of active semi-squares. Thus, the following cases have to be checked:

(a) Can any other active interval be added to the candidate clique? Then it is not a maximal clique within the set of active semi-squares and must not be added to *Q*.

(b) After all cliques *C *in *Q *have been checked and all candidate cliques built: Is there already a clique in *Q *that is identical to the candidate clique? Then it must not be added to *Q*. Are there two or more candidate cliques that are identical? Only one of them can be added to the *y*-structure.

3. If after these steps none of the cliques in *Q *contain the newborn interval *X*, it is the first element of a new clique which is added to *Q*.

If the event signals the **death **of a semi-square, it has to be deleted from every clique *C *it is an element of. If any of these cliques has been increased by the addition of a semi-square since the last deletion of a semi-square, it is a maximal clique and has to be printed out before the semi-square is deleted. Else, the semi-square is just deleted from *C*, without printing out the clique. A deletion can yield a reduced clique that is not unique anymore, or that is only a subset of some other clique in *Q*. Thus, for every clique in which some semi-square has been deleted, one has to verify its uniqueness and extensibility, and it will be deleted from *Q *if it is not unique or if it is extendable. Note that after checking these two properties, the *y*-structure invariance holds, because the *y*-structure just holds all maximal cliques of **active **semi-squares. Thus, not every maximal clique in the *y*-structure is a maximal clique with respect to the whole set of semi-squares.

**Figure 4:**
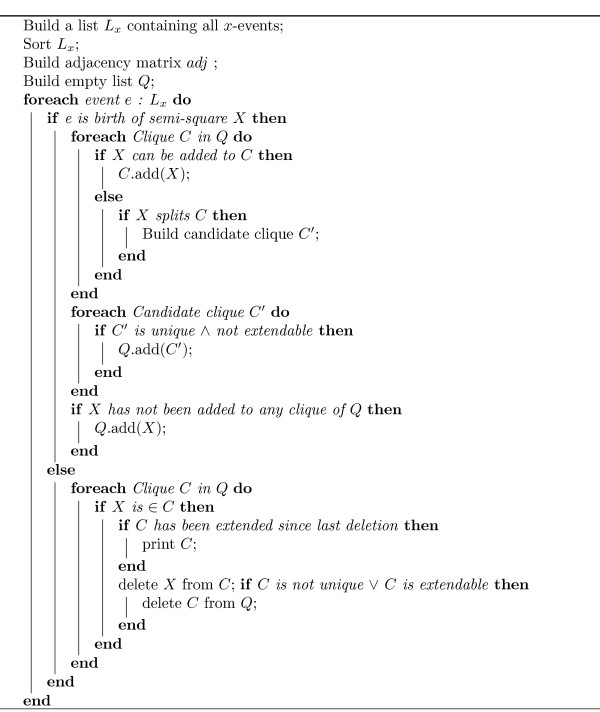
Algorithm for computing all maximal cliques.

The *y*-structure suffices to guarantee the correctness of this algorithm, because it guarantees that all maximal cliques of active semi-squares are kept in *Q*. Since a semi-square *X *is only non-active if it has not yet begun or if the rest of its corresponding interval is not long enough for any new semi-square to overlap it enough, it cannot be an element of a clique when it is non-active. For its active time, the *y*-structure guarantees that all its maximal cliques are contained in *Q*. This algorithm is thus correct because the *y*-structure invariance is maintained in all steps. The pseudocode is sketched in Fig. 4.

### Runtime analysis

There are exactly 2*n *x-events that are sorted in *O*(*n *log *n*). For every birth of a semi-square *X *we have to check all cliques in *Q *whether they contain at least one semi-square that is intersecting with *X*. In the worst case, the *y*-structure can hold up to *O*(*n*^3^) cliques (see Lemma 1) with *O*(*n*) elements each. For the further analysis we will denote the maximal number of cliques in the *y*-structure by |*Q*|. Checking whether *X *can be added to a clique or building a candidate clique can thus be done in *O*(n * |*Q*|) for all cliques in *Q*. Since every newborn semi-square can either be added to a clique, split it, or not intersect any of its elements, there can be at most *O*(|*Q*|) candidate cliques with size *O*(*n*). For each of them we have to check whether it is extendable by one of the active semi-squares and whether there is a second candidate clique that is equal to that one. To check for equality we have to match each candidate clique with each other candidate clique which takes *O*(|*Q*|^2 ^* *n*^2^) in the naive approach. To check for extensibility we have to test each active semi-square whether it can extend the candidate clique. This can be done in *O*(|*Q*| * *n*), naively. This results in a theoretical runtime of *O*(|*Q*|^2 ^* *n*^2^) per birth. For death events, the clique has to be printed if it is a maximal clique. Altogether the size of *out *is equal to the number of symbols to print all maximal cliques. Since there are at most *n *elements per clique, the size of *out *is *O*(*n*^4^). Furthermore, the deletion of a semi-square in a clique can result in a clique that is no longer maximal with respect to all active semi-squares or that is equal to another clique in the *y*-structure. Thus, we have to check for equality and extensibility again. The clique will be deleted from the *y*-structure if its size is 0, if it is equal to some other clique in the *y*-structure, or if it is extendable by one of the other active semi-squares. Also this step requires a runtime of *O*(|*Q*|^2 ^* *n*^2^). The theoretical runtime for the whole algorithm is thus given by *O*(|*Q*|^2 ^* *n*^3 ^+ *out*), which is in the worst case *O*(*n*^9^).

We will now present some small improvements in the data structure that help to reduce the work for most real data sets. First, as a fast look-up we will compute the adjacency matrix *adj *for the intersection of all pairs of semi-squares in *O*(*n*^2^).

**Figure 5:**
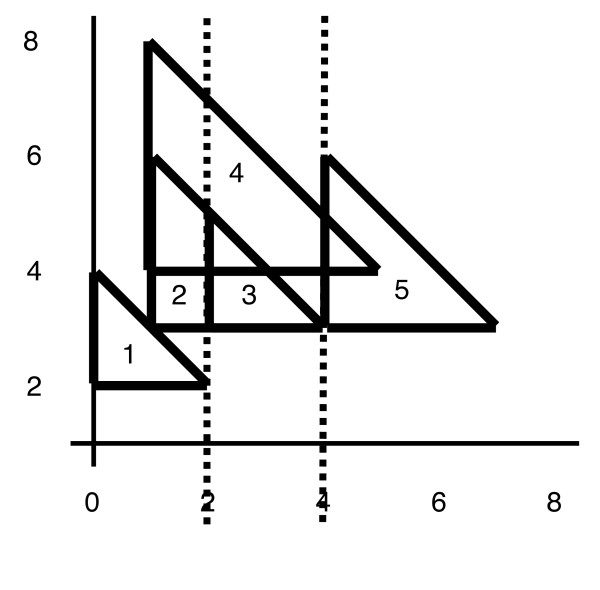
Clique {2, 3,4} at *x *= 2 has a global ordering of *B*(2), *B*(3), *B*(4), *H*(2), *H*(3), *H*(4) and a global ordering of *B*(2), *B*(3), *H*(2), *H*(3), *B*(4), *H*(4) at *x *= 4. When an interval is added to the *y*-structure it can either intersect all of the bases of a clique, or only a part of the bases, and/or all of the hypotenuses, or just a part of them. If it intersects all bases and/or hypotenuses, it can simply be added to that clique, if it only intersects a part of the bases and/or hypotenuses, it will split the clique.

As we noted before, each semi-square can be characterized by *y*_*i *_+ *x*_*i*_, the diagonal on which the hypotenuse lies. A clique *C *is now represented by two ordered lists: *H*(*C*), a list of lists of semi-squares for each diagonal on which at least one semi-square has its hypotenuse, and *B*(*C*) a list of lists of semi-squares for each *y*-coordinate on which at least one basis of any semi-square in the clique is positioned. A clique at a given *x*-position is now an assembly of ordered hypotenuses and bases as depicted in Fig. 5. A new semi-square *X *can intersect the set of bases and hypotenuses in the following ways:

1. *X *intersects none of the bases and none of the hypotenuses. Since the bases are ordered, this only occurs if the upper point of *X *is lower than the lowest basis, or if the basis of *X *is higher than the highest hypotenuse. This can be calculated in *O*(1) because *B*(*C*) and *H*(*C*) are sorted. This results in a runtime of *O*(|*Q*|) for all cliques per birth event.

2. *X *intersects either all bases and/or all hypotenuses. This is the case if *X'*s basis is lower than the lowest basis in *C *and *X'*s upper point is higher than the highest basis, and/or *X'*s basis is lower than the lowest hypotenuse and its upper point is higher than *C'*s highest hypotenuse. Both can be checked in *O*(1). If this is the case, *X *has just to be added to *C*. To insert *X'*s basis and hypotenuse at the correct place, a binary search with *X'*s height of the basis and the height of its hypotenuse is accomplished in *O*(log *n*) for every clique it is added to, i.e. with a runtime of at most *O*(|*Q*| log *n*) per birth event.

3. *X *neither intersects all bases nor all hypotenuses, but at least one basis or hypotenuse in *C*. In this case, *X *will split the clique. It is easiest to just run through both lists, *B*(*C*) and *H*(*C*), and look in *adj *which semi-squares of *C *and *X *are intersecting. By running through the ordered lists, we can just add all intersecting semi-squares in the same order to the new candidate clique, maintaining the correct sorting. Each clique has at most *n *elements, look-up of adjacency is done in *O*(1) and adding to the list is also done in *O*(1) per intersecting semi-square. Last, *X *has to be inserted into the candidate clique, again in *O*(log *n*). Thus, generating a candidate clique can be done in *O*(*n*), resulting in |*Q*| * *n *for all cliques per birth event. To find out whether this candidate clique *C' *is maximal we use the following trick which is quite fast: Given the semi-square *X*_*s *_representing the shortest interval, calculate the set of all active semi-squares that are not in *C' *but do intersect *X*_*s*_. This set *Z *can be determined in *O*(*n*) by checking *X'*_*s *_row in *adj*. Then we have to examine the extensibility of *C' *for only those semi-squares in *Z*. This takes *O*(*n*^2^), resulting in *O*(|*Q*| * *n*^2^) for every birth event. The check for equality is also quite cheap, because *Q *can again be ordered by for example the height of the lowest basis in a clique. This equality search for any other clique in the *y*-structure with the same height of the lowest basis can be done by a binary search in *O*(|*Q*| log |*Q*|). Since all cliques are ordered, a check on equality can be done in *O*(*n*), resulting in *O*(|*Q*| * *n*). Inserting the clique in the correct place in *Q *can again be done in *O*(|*Q*| log |*Q*|).

Again, the deletion of a semi-square will result in an addend of *out *to the runtime, and we have to check for equality and extensibility. Thus, our theoretical runtime is *O*(|*Q*| * *n*^3^+ *out*) which in the worst case is given by *O*(*n*^6^). Note that for real data sets this theoretical runtime will not be reached: First of all, intervals have very different lengths and therefore not all of them will be active at the same time. Second, most of the time the set *Z *of candidates for the extensibility test is very small. The same is true for the set of cliques with the same height of their lowest basis. Since the test for extensibility and uniqueness are the most expensive steps in the algorithm, this shows why the implementation can be so fast in real data sets, as will be demonstrated in section 5. But first we will give a more elaborated algorithm that shows that the runtime complexity of finding all maximal cliques in *c*-max-tolerance graphs is considerably lower than the corresponding runtime in max-tolerance graphs.

## 4 An efficient output-sensitive algorithm to determine all maximal cliques in *c*-max-tolerance graphs

### 4.1 Introductory discussion

We shortly recall the *O*(*n*^3 ^+ *out*) algorithm from [9]. In this algorithm, it has been shown that each maximal clique is uniquely described by the 3 parameters *t, h *and *v *denoting the hypotenuse *t *of the lowest semi-square, the highest base *h *of any semi-square and the rightmost vertical side *v*. The drawback of the algorithm given in [9] is that it needs *O*(*n*^3^) time even if there are only very few maximal cliques. In the case of *c*-max-tolerance graphs we can now present a considerably improved output-sensitive algorithm.

Our description consists of two steps: First we will give an algorithm that computes all candidates for maximal cliques with one fixed parameter, say a given *lowest *hypotenuse *t*. We show that all maximal cliques with fixed parameter *t *can be determined in time *O*(*n *log *n *+ *out*) where *out *is the size of the output.

The problem is, however, that these maximal cliques could still be extendable by a semi-square with an even lower hypotenuse *t'*. Thus, such a maximal clique with fixed parameter *t *is only a *candidate *that has to be checked for extensibility before printing it out. In the second step, we will show how to avoid the computation of candidates that do not represent maximal cliques such that our final algorithm will truly be output-sensitive.

### 4.2 Maximal cliques regarding parameter *t*

Let *t *be the hypotenuse of the lowest semi-square in the cliques, and let *r *(*t*) denote the whole square defined by the diagonal *t*. Note that it makes sense to use *t *in the two different but related contexts. Using the same notation as in [9], *P*(*t*) denotes the set of semi-squares that include the left endpoint of *t*, *Q*(*t*) is the set of semi-squares that include *t'*s right endpoint, and *R *(*t*) denotes the set of semi-squares that intersect *t *but include none of its endpoints. *P*^*s*^(*t*), *Q*^*s*^(*t*), and *R*^*s*^(*t*) denote the set of the full squares corresponding to the set of semi-squares *P*(*t*), *Q*(*t*) and *R *(*t*), while more importantly, we will consider the sets *P*^*r*^(*t*), *Q*^*r*^(*t*) and *R*^*r*^(*t*) which denote the set of rectangles given by the intersection of *r *(*t*) with each single element of *P*^*s*^(*t*), *Q*^*s*^(*t*) and *R*^*s*^(*t*).

Note that the left upper corner of *r *(*t*) is also the left upper corner for the rectangles in *P*^*r*^(*t*), while the right lower corners are the same as that of the corresponding semi-squares in *P*(*t*). Similar facts hold for rectangles in *Q*^*r*^(*t*) and *R*^*r*^(*t*).

The following observation provides the idea to how to determine the maximal cliques with lowest semi-square *r *(*t*): Each point *x *∈ *r *(*t*) is overlapped by a set of rectangles from *P*^*r*^(*t*) ⋃ *Q*^*r*^(*t*) ⋃ *R*^*r*^(t). The **crucial observation **is that their corresponding semi-squares are also pairwise intersecting, so *x *∈ *r *(*t*) denotes a clique of semi-squares. This is important, because we state here that in the restriction to *r *(*t*) we have an equivalence between the intersection of the rectangles and the intersection of the semi-squares. Thus, the question to finding maximal cliques in the *c*-max-tolerance graph is now reduced to finding an area where a maximal set of rectangles intersects.

**Figure 6:**
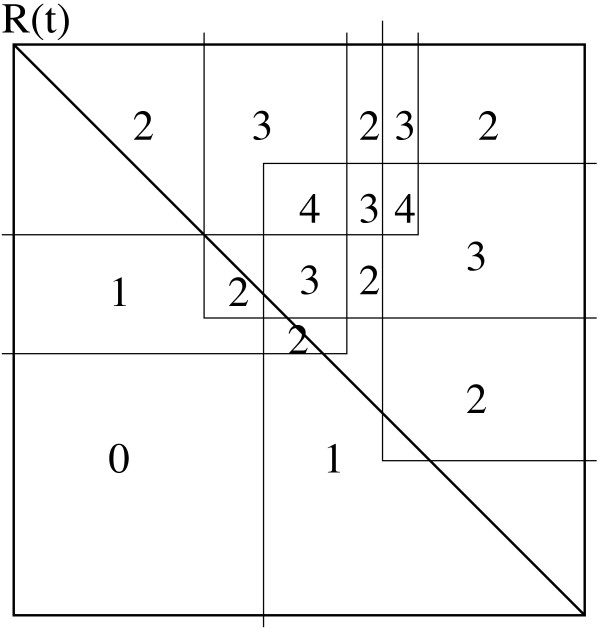
Computing the maximal cliques regarding the hypotenuse *t *by intersection *r *(*t*) with *P*^*r*^(*t*),*Q*^*r*^(*t*) and *R*^*r*^(*t*). In the example, we have two rectangles from *P*^*r*^(*t*), two from *R*^*r*^(*t*) and one from *Q*(*t*). The numbers denote the cover-values of the corresponding polygons.

The points intersected by the same set of rectangles form simple orthogonal connected polygons *p*. Each such polygon *p *is also characterized by the cardinality of this set which we call *cover *(*p*). Polygonal regions *p' *adjacent to *p *have either an additional intersection by another rectangle or one intersection missing. So the cover-variables of adjacent polygons differ by exactly 1 (see Fig. 6). If all adjacent polygons have a lower cover-parameter than *cover *(*p*), *p *denotes a maximal clique. We call *p locally maximal *in this case.

**Lemma 3 ***Locally maximal polygons in r *(*t*) *exactly determine the maximal cliques with lowest hypotenuse t*.

#### The algorithm

We perform a left-to-right sweep. As the underlying data structure we keep the list *L *of all polygons which are currently intersected by the sweepline as well as a distinct list *L*_*M *_of all locally maximal polygons. We start our sweep at the left side of *r *(*t*) initializing list *L *by the polygons defined by all rectangles of *P*^*r*^(*t*) in increasing order of their lower boundaries. *L*_*M *_is initialized with the topmost polygon representing the intersection of all rectangles in *P*^*r*^(*t*). Two basic events occur while sweeping from left to right:

1. A rectangle from *P*^*r*^(*t*) ends

2. A rectangle from *Q*^*r*^(*t*) or *R*^*r*^(*t*) is added.

1. Let *s *be the rectangle from *P*^*r*^(*t*) with the right lower corner *c *that ends. Since *s *intersects all polygons from *c *up to the upper boundary or *r *(*t*), the removal of *s *decreases the cover-variables by one for all polygons above *c*, the two polygons adjacent to *c *have to join, and the maximal polygons above *c *have to be output. The join-operation can be done by updating list *L *after locating the two polygons by binary search for *c*. The output operation can be performed easily by scanning list *L*_*M *_from the top until the *y*-coordinate of *c *has been reached.

Note that there will not arise any new local maxima, and all previous maxima remain. One important speciality is that the maxima we just output should not be output even they still represent maxima. We call them false maxima, we remove them from the list *L*_*M *_and insert them in a list *L*_*F *_ordered by their *y*-coordinates. Note that false maxima have to be reinserted into list *L*_*M *_again as soon as they are covered by a new rectangle from either *Q*^*r*^(*t*) or *R*^*r*^(*t*). This might happen as described in the next case:

2. A rectangle *s *from *Q*^*r*^(*t*) ⋃ *R*^*r*^(*t*) starts to be intersected by the sweepline. We discuss the case that *s *∈ *Q*^*r*^(*t*), the other case is symmetrical.

Let *c *be the left upper corner of rectangle *s. s *adds a new intersection to all polygons below *c*, the polygon containing *c *is split into two and the cover-variables of all of them increase by one. Note that a new locally maximal polygon might arise below *c*. All false maxima below *c *become 'true' maxima again. They are deleted from *L*_*F *_and inserted again into *L*_*M*_. The operation can be performed by locating the polygon to be split by binary search in *L*, scanning the list *L*_*F *_until the *y*-coordinate of *c *is reached, and inserting all false maxima back into *L*_*M*_.

Analysis: At each event we have to perform a binary search for corner *c*. A direct implementation includes the insertion and deletion of maxima and false maxima into the corresponding lists in time *O*(log *n*). Finally it leads to a runtime of *O*(*n *log *n *+ *C*(*t*) log *n *+ *out*), where *C*(*t*) denotes the number of maximal cliques with lowest hypotenuse *t*.

Next we will show how to improve the efficiency of the algorithm to *O*(*n *log *n *+ *out*): Instead of two separated lists *L*_*M *_and *L*_*F *_we keep only one doubly linked list *L*_*D *_of interleaved blocks containing false maximals and 'true' maximals ordered by *y*-coordinates. A block denotes a maximal sequence of maxima of one or the other kind. We keep also the blocks internally connected.

Each block is created by a certain event and it will be removed eventually. We count only the number of block creations, which naturally links the number of block removals, and show that only *O*(*n *+ *C*(*t*)) blocks will be created.

One possible event is the creation/removal of a polygon adjacent to corner *c *of a single maximum in the list *L*_*D*_. Possibly one new block is created by splitting an old block into two. After having located the position of the block, corresponding to the *y*-coordinate of *c*, by binary search, this can be done in constant time. Clearly there are at most *O*(*n*) such events.

All other events consist of browsing through the lists of blocks starting from the upper or lower boundary of rectangle *r *(*t*), output the contents and join adjacent blocks until reaching the polygon with *y*-coordinate of corner *c*. Hence in these events, each operation creates one or two new blocks, but might remove *k *blocks in time *O*(*k*).

In total, we are using only time *O*(*n *log *n*) time.

**Lemma 4 ***Determining the maximal cliques with lowest parameter t takes time O*(*n *log *n *+ *out*).

### 4.3 Avoiding false maximal cliques when computing candidates for hypotenuse *t*

In the previous subsection, we have described the efficient computation of all maximal cliques having a specific triangle *t *as their lowest element. To provide truly output-sensitive algorithms we have to notice that some of those cliques, say M, might not be maximal overall, since there might be a triangle *s*(*t'*) with hypotenuse *t' *such that *M' *= *M *⋃ *s*(*t'*) is also a clique. Clearly *t' *is lower than *t*. Note that *M' *will be found when computing the maximal cliques with lowest element *t'*.

So, when considering *t *we have to avoid those cliques, which are not truly maximal.

#### 4.3.1 The intersection staircase

The first idea is to compute for rectangle *r *(*t*) the intersection of *r *(*t*) with all *r *(*t'*) where *t' *does not belong to *P*(*t*) ⋃ *Q*(*t*) ⋃ *R *(*t*). Clearly, polygons in *r *(*t'*) ⋂ *r *(*t*) might represent cliques which are maximal in *r *(*t'*) but are false maximal in *r *(*t*) since *t' *has not been considered there. To neglect the area where such rectangles *r *(*t'*) intersect *r *(*t*) seems to be a good first step, although it will turn out that this is not sufficient. The union of those intersections is determined by a set of maximal rectangles, which form a kind of a staircase pattern above the diagonal of *r *(*t*) which we call the intersection staircase. The intersection staircase will be represented by a list of the right upper endpoints with decreasing *y*-coordinates and increasing *x*-coordinates.

The computation of the intersection staircase can be done using a list of the rectangles *r *(*t'*) decreasingly ordered according to the *y*-coordinate of their upper boundaries. For each rectangle *r *(*t'*) we check if it intersects the diagonal of *r *(*t*) and does not belong to *P*(*t*) ⋃ *Q*(*t*) ⋃ *R *(*t*). If the right boundary of the last element of the actual intersection staircase is also intersected by the upper boundary of *r *(*t'*) or if the last element of the intersection staircase ends above *r *(*t'*), *r *(*t'*) is appended to the list of the intersection staircase.

**Figure 7:**
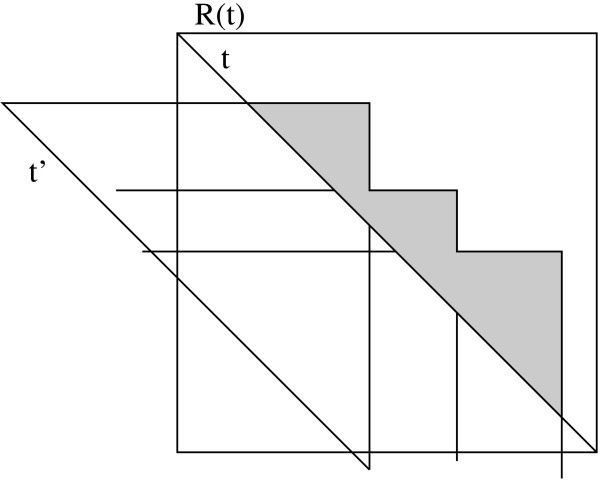
The grey area denotes the basic intersection staircase, which describes the maximal cliques which potentially must not be considered since they have been considered already during the computation of the maximal cliques for hypotenuses like *t' *lower than *t*. In the example, we have three diagonals *t' *lower than *t *whose rectangles *r *(*t'*) intersect *t*.

Clearly, the intersection staircase can be computed in time *O*(*n *log *n*). Unfortunately, this is not sufficient, since there might be rectangles *r *(*t*") ∈ *P*^*r*^(*t*) ⋃ *Q*^*r*^(*t*) ⋃ *R*^*r*^(*t*) which intersect *r *(*t'*) but not the diagonal *t' *of *r *(*t'*), hence they have not been taken into account. Clearly, the intersection of *r *(*t*") with *r *(*t*) ⋂ *r *(*t'*) has to be considered if it infers new cliques which have not been found when processing *r *(*t'*). Hence the intersection staircase which defines the forbidden area when processing *r *(*t*) has to be refined and its area will decrease (see Fig. 7).

#### 4.3.2 Refinement of the staircase

We keep the intersection staircase as an ordered list of right upper corners of the corresponding rectangles *r *(*t'*). First we describe how rectangles from *P*(*t*) influence the intersection staircase.

**Figure 8:**
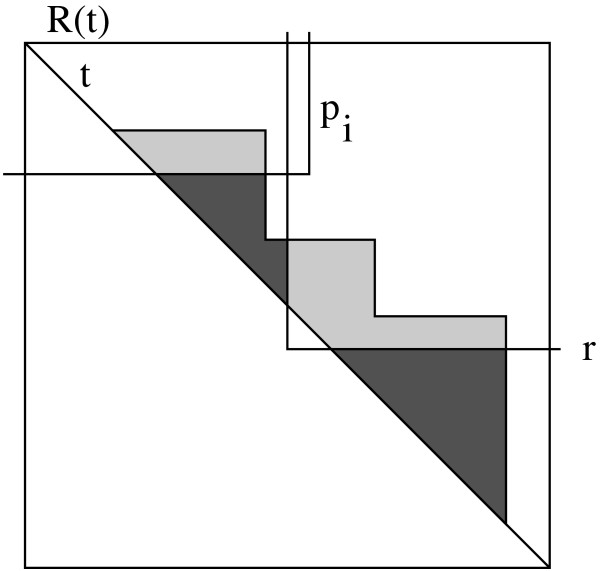
The basic intersection staircase shown in grey must be refined since there are rectangles like *Pi *∈ *P*^*r*^(*t*) and *r *∈ *R*^*r*^(*t*) which have not been considered before in the runs for *t' *and which reduce the basic intersection staircase such that a lower staircase shown in darkgrey remains. Hence for the computation of the maximal cliques regarding *t *we have to reduce the area of the upper half only by the refined staircase.

We consider all the rectangles of *P*(*t*) and how they intersect the staircase. On this behalf, we sort the lower left corners *a*_*i *_of the rectangles *p*_*i *_according to the difference between *y*- and *x*-coordinates and process the rectangles in this ordering. Clearly, all those corners lie to the left of the left boundary of *r *(*t*). Assume that we have already processed the corners *a*_1_,..., *a*_*i*-1 _as well as the topmost *j *steps of the staircase with *j *≥ 0. Let *s*(*t*_*j*_) be the actual step defined by rectangle *r *(*t*_*j*_) to be considered. We consider *p*_*i*_. If *p*_*i *_is below of the diagonal of *r *(*t*_*j*_), it can be neglected since it has been considered while processing *r *(*t*_*j*_). Otherwise, if the lower boundary intersects step *s*(*t*_*j*_), the upper part of *s*(*t*_*j*_) is cut off, and we proceed to *P*_*i*+1_. If the lower boundary is even lower than the whole step, *s*(*t*_*j*_) is completely cut off, and we proceed to *s*(*t*_*j*+1_). In this case, we reconsider *pi *(see Fig. 8).

Clearly, since there are at most *n *points *p*_*i *_and steps *s*(*t*_*j*_), the whole process takes *O*(*n *log *n*).

Analogously, we can refine the intersection staircase by considering the rectangles in *Q*(*t*), which have not been considered in the staircase-defining rectangles *r *(*t'*).

For rectangles in *R *(*t*), the refinement is slightly different, so we will consider this in more detail:

The intersection staircase now separates the area representing cliques that have not been computed before from those which either have been computed before or which have not been assigned yet. Hence we only move the intersection staircase downwards!

The intersection staircase consists of a list of points *c*_1_,...,*c*_*k *_which are not necessarily corners of rectangles, but at least they can uniquely be assigned to rectangles *r *(*t'*) ∌ *P*^*r*^(*t*) ⋃ *Q*^*r*^(*t*) ∈ *R*^*r*^(*t*). Assume that these points are ordered with decreasing *y*-coordinates. We denote the corresponding diagonals *t*_1_,...,*t*_*k *_analogously.

First we state an important observation:

**Lemma 5 ***Only rectangles r *(*t'*) *which are part of the intersection staircase have maximal diagonals. Hence the non-maximal r *(*t'*) *that is not in P*(*t*) ⋃ *Q*(*t*) ⋃ *R *(*t*) *does not need to be considered*.

The elements of *R*^*r*^(*t*) are similarly ordered according to the difference between *y*- and *x*-coordinate of their left lower corner. The idea is that when comparing a rectangle *r *(*t'*) from the staircase with an element *s *from *R*^*r*^(*t*) then the staircase should not be changed if *s *intersects *t'*. Otherwise, the intersection of *s *must be cut off from the staircase.

More formally, we start with the topmost step of the staircase and let *t' *be the corresponding diagonal. Let *s*_*max *_be the first element in the ordered list from *R*^*r*^(*t*). If the left lower corner is below *t'*, then *s*_*max *_has been considered already in the computation of the maximal cliques regarding *t'*. Hence it can be disregarded and it does not change the staircase. We can delete *s*_*max *_from the list and let the next element be s_*max*_.

If the left lower corner of *s*_*max *_is above *t'*, we compute the intersection of *s*_*max *_with the actual step of the staircase, and remove it either completely or only partially depending on the size of the intersection. In the first case, we proceed to the next lower step of the staircase while in the second case, we remove *s*_*max *_from the list and get a new *s*_*max*_.

In all cases, the operation can be done in *O*(1) time, and in total we have only *O*(*n*) operations. This concludes the description how we effectively restrict the area of *r *(*t*) to be considered for computation of just those maximal cliques which have not been computed before.

Hence we can summarize the whole section by

**Theorem 1 ***In time O*(*n*^2 ^log n + *out*) *we can determine all maximal cliques in c-max-tolerance graphs*.

Note that the naive bound of *O*(*n*^4^) for *out *can be improved to *O*(*n*^3^) by writing down only the differences between subsequent maximal cliques. This is supported by the above methods to determine the maximal cliques in a plane sweep approach.

## 5 Experiments

This section shows some results on using our implementation for finding all maximal cliques in artificial and biological data sets.

### Runtime on artificial and biological datasets

In this section we want to demonstrate how fast the implementation of our simple algorithm is. We compare the runtime to that of Cliquer [8]. For the test we use both artificial as well as biological data sets. The artificial datasets were generated in the following way: For a given interval of some maximal length ℓ_*max *_we computed all possible sub-intervals within this interval. From this set of possible sub-intervals, a given percentage number of sub-intervals was chosen uniformly at random for the further evaluations. Note that the number of possible sub-intervals in an interval with length ℓ is roughly ℓ^2^, and thus the number of maximal cliques is *O*(ℓ^6^). We chose two values for ℓ_*max*_, namely 20 and 100. Then, the overall runtime to calculate all maximal cliques was determined for all constraints within 0.05 ≤ *c *≤ 0.95 in steps of 0.05. For Cliquer we could only use an interval of length ℓ_*max *_= 20 and a randomly chosen set of 30% of all possible sub-intervals. For other test sets Cliquer had runtimes longer than many hours or even days. For ℓ_*max *_= 20 and *c *= 0.05 Cliquer took nearly three hours to compute all maximal cliques, for *c *= 0.1 it took nearly one hour to compute all maximal cliques, for *c *= 0.15, *c *= 0.2, *c *= 0.25 it took approximately 8, 4, and 1 minutes, respectively. For all *c *≥ 0.3 it took under a minute to compute all maximal cliques and it took below 0.01 seconds for all *c *≥ 0.5. Our implementation needed no more than 50 ms for any of the *c*-values.

**Figure 9:**
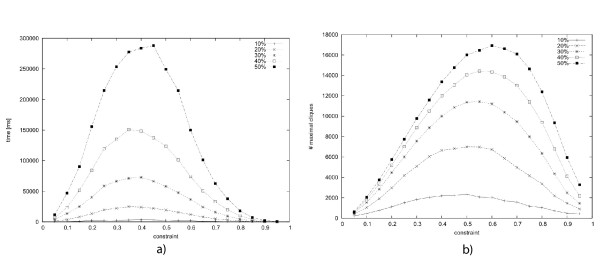
**a) **The runtimes shown in this figure are based on artificial data sets. Here, the maximal length is 100 and from all possible intervals a random set is chosen, ranging from 10% to 50% of all possible intervals. The time needed to compute all maximal cliques for all constraints from 0.05 to 0.95 is given in *ms*. **b) **gives the number of maximal cliques found in the data sets.

A second experiment was made on a much larger data set where the interval had a length of ℓ_*max *_= 100. We used 10%, 20%, 30%, 40% and 50% of the total set of possible sub-intervals chosen uniformly at random. Again, Cliquer could not compute the maximal cliques for any of the data sets in any reasonable time. The results for the runtimes of our algorithm for these data sets are visualized in Fig. 9. Note that the data sets contained 1000, 2000, 3000, 4000 and 5000 intervals, respectively. We see that in all cases and for all chosen constraint values *c*, the runtimes were below 30 seconds. Even for the largest data set with 5000 sub-intervals the computation of the full statistics needed less than 4 minutes.

It is an interesting observation that the runtime seems to follow the number of cliques in the set, in contrast to the runtime of the Cliquer that is mainly determined by how interwoven the cliques are. The runtime of our algorithm thus indicates that for these random data sets the runtime is bound by *out*. Biological data sets are very different in structure from random data sets, which is caused by the clusteredness of their sequences. Since genes are often composites of different functional domains, aligned sequences have a high probability to center around those domains and build groups of 'similar' sequences with respect to the position and length of their alignment to the query.

For the test of our algorithm on biological sequences three BLAST-derived data sets were chosen as follows: From a bacterial artificial chromosome (BAC) and fosmid library of the genome of *Pristionchus pacificus *[13] that contains 78690 sequences, we reconstructed a genetic element that we characterized as a transposon from the maT family (S. Steigele, unpublished). Experimental hybridisation assays proved that this transposon is highly repetitive in the *Pristionchus pacificus *genome (A. Breit, unpublished). A BLASTN search (E-value < 10^-6^) of this transposon against all sequences of the BAC and fosmid library was conducted, resulting in 126 hits below the E-value threshold. We refer to this data set by *PpmaT*. Furthermore, two multi-domain proteins, both containing repeated domains, were subject to BLASTP searches (E-value < 10^-6^) versus the UniProt database [11]. The protein SH3 and multiple ankyrin repeat domains protein 1, short Shank1, contains 7 ANK repeats, 1 PDZ domain, 1 SAM domain and 1 SH3 domain. The BLAST search resulted in 86 hits below the E-value threshold. We refer to this data set by *Shank1*.

**Figure 10:**
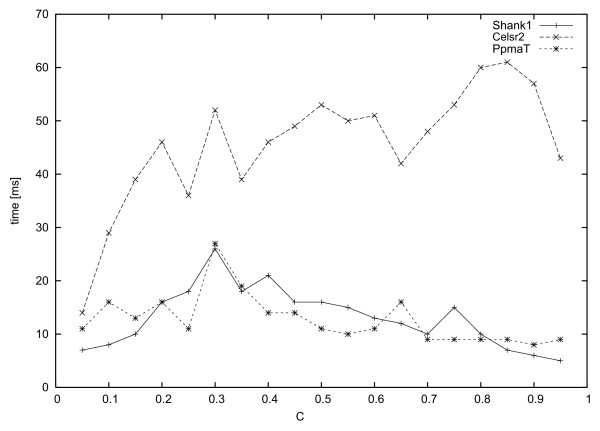
All maximal cliques in three different biological data sets were computed. The time needed by our algorithm is given in ms in dependence of the chosen constraint value *c*.

The protein Cadherin EGF LAG seven-pass G-type receptor 2, short Celsr2, is a receptor that may have an important role in cell-cell signaling during nervous system formation. It belongs to the G-protein coupled receptor 2 family, LN-TM7 subfamily, and contains 4 cadherin domains, 7 EGF-like domains, 1 GPS domain, 1 laminin EGF-like domain, 2 laminin G-like domains and 1 transmembrane receptor domain. The BLAST search resulted in 249 hits below the E-value threshold. We refer to this data set by *Celsr2*. Fig. 10 shows the times needed to compute all maximal cliques for the given data sets for all *c *between 0.05 and 0.95 in steps of 0.05. Generally we observe in all three cases that the runtime within one data set is almost independent of the constraint value *c*, differing only by a factor of about 2 across each curve. For all cases runtime was well below one second, while Cliquer needed for some of the *c*-values many hours and even longer. We have therefore again not presented Cliquer's runtime results for these data sets. The computation of the full curves needed less than one second.

**Figure 11:**
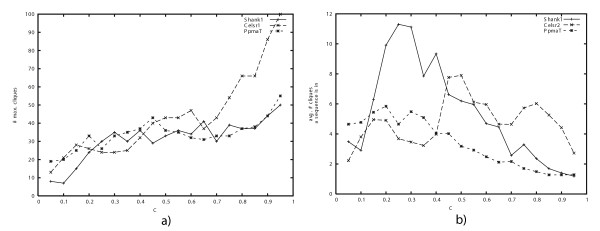
**a) **The number of maximal cliques found and **b) **the average number of maximal cliques a sequence is contained in are highly sensitive on the chosen *c*-value as can be seen for three biological data sets.

For the same data sets, Fig. 11a) shows the number of cliques found at these constraint values and Fig 11b) shows the average number of cliques a sequence is member of. In comparison to the artificially derived data sets, runtime in these cases seems to be more dependent on the input size than on the number of maximal cliques. Furthermore, there is also no obvious dependency between the chosen *c*-value and the number of maximal cliques. We also observe for quite a large range of constraint values *c *on average a sequence is contained in up to 30% of all maximal cliques. Even for large *c*-values (*c *> 0.6) on average a sequence is contained in up to 10% of the maximal cliques.

### Computing a partition of *S *from maximal cliques

Since the membership of a sequence is almost never unambiguous, a heuristic is needed in order to partition the set of sequences into meaningful clusters. The set of all maximal cliques for a given constraint *c *guarantees us that every two members of a maximal clique will at least share a part of their sequence of length (|*I*_*i*_| * *c*). Let *I*_*c*_(*C*_*i*_) denote that part of the query sequence that is overlapped by all sequences in clique *C*_*i*_, i.e.,

*I*_*c*_(*C*_*i*_) = [max {*x*_*j*_|*I*_*j *_∈ *C*_*i*_}, min {*y*_*j*_|*I*_*j *_∈ *C*_*i*_}]       (6)

**Figure 12:**
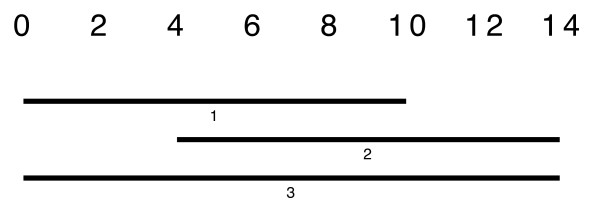
With a constraint of 0.6 intervals 1, 2, 3 build a clique. The common overlap of all 3 intervals is [4-10], which is only 3/7 of interval 3.

It is an important observation that this common part of the sequence does not have to overlap each sequence in *C*_*i *_by c* 100%, as is shown in Fig. 12. If now a sequence *X *is a member of many maximal cliques, it should be assigned to that clique *C*_*i *_whose shared sequence part *I*_*c*_(*C*_*i*_) overlaps *X *best, i.e. is maximal.

**Figure 13:**
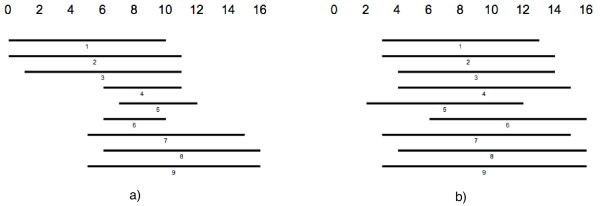
**a) **There are three distinguishable clusters: {1, 2, 3}, {4, 5, 6}, {7, 8, 9} but as long as *c *is below 4/11 there is only one maximal clique embracing all intervals. Not until *c *is larger than 3/5, the wanted three sets will emerge as maximal cliques. **b) **Here, the situation is different. Intuitively, only one set embracing all intervals should emerge. But this homogenous set will be split into two maximal cliques when *c *is greater than 3/5: one contains interval 5 and one interval 6.

The other problem is that the number and structure of maximal cliques is of course depending on the chosen constraint value *c*. There are two opposing situations that make it hard to find the best *c*-value: The first is sketched in Fig 13a) where three different clusters of sequences are visible, but they will only be found if *c *is larger than 0.6. In Fig. 13b) there is only one cluster, but if *c *is larger than 0.6 then two maximal cliques will emerge.

Thus, we have two contradicting goals: To get a higher specificity of the maximal cliques it is good to use a large *c*-value. But if it is too large it will split 'good' cliques into too many parts. Our aim was to find a heuristic that is based on a *c*-value that is small enough as not to split 'good' clusters but can find subsets of the maximal cliques that are more specific than the whole clique.

For this, the following heuristic is used:

1. Add all maximal cliques to a list *L*.

2. For all cliques *C *in *L *compute the shared overlap interval *I*_*C*_, i.e., the maximal interval that is overlapped by all of the intervals in the clique.

3. For every interval *I *that is in more than one clique, compute the overlap between *I*_*C *_and *I *and multiply this value with the number of intervals in this clique.

4. Assign every interval *I *to that clique where the product of overlap with *I*_*C *_and number of intervals in that clique is maximal.

This basic variant yields good results but it can be improved by the following procedure: For every pair *A, B *of cliques compute the intersection *A *⋂ *B*. If there are many intervals in this intersection set, e.g., |*A *⋂ *B*| > 3, and only a few intervals that are not, the intersection of the two cliques represents intervals that are very similar to each other. These intervals would be in one clique if the *c*-value would have been more restrictive, i.e., larger. But due to the low *c*-value, there are other intervals to the left and right of the intervals in the intersection set that interact with the intervals in *A *⋂ *B *but not with each other. As discussed above, a low *c*-value has the advantage of keeping good clusters together, and thus *c *should not be too high in the beginning. However, the effect of a larger *c*-value can be mimicked by adding those intersection sets to the list *L *in the first step of the above described mechanism and to allow the intervals to assign them to any set in *L *be it a clique or an intersection set.

The given heuristic guarantees that every two intervals in a set tolerate each other, because it is built on the maximal cliques of tolerating intervals. The decision of membership has been built on a composite of the overlap with the shared sequence and the size of the set to reduce the number of sets globally. The size of a set is of course volatile because not all of its designated members will finally be assigned to it. But in each case the overlap with the shared sequence of any set will never decrease if another member is deleted from the set, so the heuristic is stable in this respect.

We have already indicated at the beginning of this article (s. Fig. 1), that many partitions of a set *S *of sequences seem to be reasonable by regarding only their begin and ends. In order to facilitate a manual revision of the calculated partition of each set of sequences these sets are displayed in the order of the diagonal of their smallest member, i.e., the sum of its *x*-position and its length.

**Figure 14:**
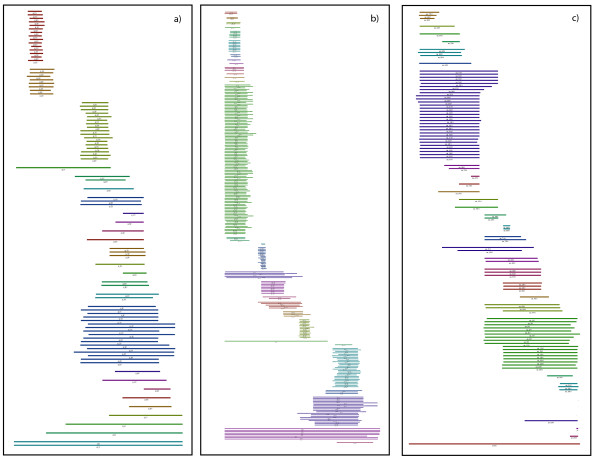
The partitions found by the heuristic described in the text, for a constraint value of *c *= 0.65. a) Shankl, b) Celsr2, c) PpmaT. Members of the same clique are colored equally.

The results of this heuristic applied to the three biological data sets are given in Fig. 14a–c. The colorization helps to identify the members of one clique. When comparing the cliques with the annotation of the input query sequence individual domains as well as combination of several domains were retrieved and corresponded almost perfectly to the annotated domains of the input protein sequence.

## 6 Discussion

BLAST is a very common algorithm in bioinformatics used for annotation of an unknown sequence or for sampling sequences from a database sharing homologous regions with the input sequence. One goal of BLAST is to identify regions of conserved DNA or protein sequence. Another goal is to detect groups of sequences that are highly similar with respect to the position and the length of conservation with the query sequence. For a given query sequence, BLAST returns the hits ordered statistical significance of each hit. When viewed, this ordering does not necessarily represent a clustering with respect to length of the hits and position in the query sequence. Thus, extraction of possible clusterings of the sequences is not always straightforward. One goal of this article was therefore to provide a method that allows an automatic clustering of sequences returned from a BLAST run, such that the user can decide whether to maximize the lengths of the common region of the sequences within a cluster or whether to maximize the size of the clusters.

In this article we have shown that finding groups from BLAST reports can be reduced to computing maximal cliques in so-called *c*-max-tolerance graphs. We have presented two algorithms that allow the enumeration of all maximal cliques in *c*-max-tolerance graphs in polynomial time. The first algorithm has an easy implementation to compute all maximal cliques in *c*-max-tolerance graphs, but it has a theoretical runtime of *O*(*n*^6^). The second algorithm is technically very elaborate, and shows that enumeration of all maximal cliques in *c*-max-tolerance graphs can be computed in a runtime of *O*(*n*^2 ^log *n *+ *out*), where *out *denotes the size of the output. The algorithm improves a result found for general max-tolerance graphs, for which the computation of all maximal cliques has a runtime of *O*(*n*^3^) [9].

Though our simple algorithm in theory has a higher runtime than the more technical one, when applied to real data, runtimes were nonetheless impressive. Comparison with the popular application Cliquer [8], another exact algorithm to compute all maximal cliques in general graphs, showed that for many instances of our test cases, Cliquer could not report results within hours, while our algorithm finished most computations within milliseconds.

The maximal cliques represent a clustering of the sequences detected with BLAST. Thus with the help of this clique-finding algorithm, clusters can now be quickly and automatically identified even from very large BLAST reports. The result of the clustering can then be further processed for example for phylogenetic reconstructions of the sequences in the clusters. At this point one might discuss the biological usefulness to compute all maximal cliques. As we have seen from the biological data sets used for our experiments, it is very common to have largely interlinked sequences, i.e., where one unique clustering and/or maximal clique does not exist. This is for example a common feature in the case of multidomain proteins, where the query protein contains say *k *domains, and BLAST reported subject proteins will show a variety of combinations of these *k *domains. A goal of applying the maximal cliques search algorithm is to cluster individual domains as well as all possible combinations of domains. The examples in figure 14 illustrate the result of these clusterings. Here one also clearly sees that other reasonable partitions are possible.

Another application for this algorithm is the prediction of gene boundaries within a genomic sequence. For this, mapping of cDNA/ESTs to a genome sequence is used. The goal of this strategy is to predict the gene boundaries and/or its exon/intron structure using cDNA/ESTs. Applying our simple maximal clique algorithm to a large test case (with more than 2800 intervals), for which a human genomic locus was compared against the EST database from the NCBI, yielded results within a few seconds with a perfect clustering of EST loci (data not shown).

The use of the *c*-value is an important parameter for biological applications. Again we think of the situation of reconstructing phylogenetic trees from sequences reported from a BLAST run. Here different *c *parameters can be used to either optimize the length of the overlaps or the size of the maximal cliques. A model where the factor *c *is not a tolerance relative to the length but a fixed constant independent of the length might be of some biological relevance. An extension towards such a model or even a mixture seems plausible.

Examples from biological data sets indicated that most of the sequences are contained in several cliques. Again, it is not straightforward to deduce a disjoint clustering of the sequences from the maximal cliques. We have described a heuristic that computes a partitioning of sequences from the set of all maximal cliques in a *c*-max-tolerance graph. This heuristic when applied to protein sequences containing several domains partitions the set of all maximal cliques into clusters, where the sequences within the clusters reflect the domain structure of the input sequence.

After cliques and clusters have been computed postprocessing such as visualization of the groups or integration into pipelines that need clustered sequence data is now very easy and automizable. And therefore we hope that the algorithms described here have further impact for the bioinformatics community.

## Availability

The implementation of the first algorithm is available on request from the first author.

## Authors' contributions

KAL implemented the simple version of the maximal cliques algorithm and performed the test runs. MK and KAL developed the efficient algorithm, proved its polynomial complexity and wrote most of the manuscript. SS retrieved the biological data and participated in the discussions. KN developed the project idea and participated in the manuscript preparation. All authors read and approved the final manuscript.
